# Socioeconomic inequalities in suicide mortality before and after the economic recession in Spain

**DOI:** 10.1186/s12889-017-4777-7

**Published:** 2017-10-04

**Authors:** Carme Borrell, Marc Marí-Dell’Olmo, Mercè Gotsens, Montse Calvo, Maica Rodríguez-Sanz, Xavier Bartoll, Santiago Esnaola

**Affiliations:** 1 Agència de Salut Pública de Barcelona, Plaça Lesseps 1, 08023 Barcelona, Spain; 2 CIBER Epidemiología y Salud Pública (CIBERESP), Madrid, Spain; 3 Institut d’Investigació Biomèdica (IIB Sant Pau), Barcelona, Spain; 4 Universitat Pompeu Fabra, Barcelona, Spain; 5 Department of Health of the Basque Country, Vitoria-Gasteiz, Spain

**Keywords:** Suicide, Mortality, Inequalities

## Abstract

**Background:**

An increase in suicide mortality is often observed in economic recessions. The objective of this study was to analyse trends in socioeconomic inequalities in suicide mortality before and during the economic recession in two geographical settings in Spain.

**Methods:**

This study analyses inequalities in mortality according to educational level during 3 different time periods based on individual data from the Basque Country and Barcelona city. We analysed suicide mortality data for all residents over 25 years of age from 2001 to 2012. Two periods before the crisis (2001–2004 and 2005–2008) and another during the crisis (2009–2012) were studied. We performed independent analyses for sex, age group, and for the two geographical settings. We fit Poisson regression models to study the relationship between educational level and mortality, and calculated the relative index of inequality (RII) and the slope index of inequality (SII) as comparative measures.

**Results:**

For men in the Basque Country, all RII values for the three time periods were similar and almost all were greater than 2; in Barcelona the RII values were generally lower. The SII values for Barcelona tended to decrease over time, whereas in the Basque Country they showed a U-shaped pattern. Among women aged 25–44 years we found an association between educational level and suicide mortality during the first time period; however, we found no clear association for other age groups or time periods.

**Conclusion:**

This study within two geographical settings in Spain shows that trends in inequalities in suicide mortality according to educational level remained stable among men before and during the economic recession.

## Background

Suicide is one of the most important causes of mortality among the young population. Several reviews argue that suicide mortality rates are higher in populations with low socioeconomic position [1–4]. In addition, some authors have found that the socioeconomic inequalities in suicide mortality increased among men, but not among women, at the turn of the twenty-first century [5, 6].

Previous studies have clearly shown that economic recession and high unemployment are associated with an increase in suicide mortality [7, 8]. Recessions result in increased debt, job loss, house eviction and reduced public spending, all of which are associated with poor mental health [9]. Nearly all European countries have experienced a marked increase in suicide mortality rates during the current economic recession, particularly among men of working age and the unemployed [10–12]. In a systematic review of 38 studies [13], 31 studies reported a positive association between economic recession and increased suicide mortality rates. Spain is one of the European countries that has suffered most during the current economic recession, with high levels of unemployment, predominantly among the young population. Several Spanish studies have shown that overall suicide mortality has generally increased [14–17], although this trend is not seen in all age groups and sexes [18].

While the increase in suicide mortality during the current economic recession has been described for several geographic locations, there is insufficient evidence on the trends of socioeconomic inequalities in suicide mortality. Our hypothesis is that these inequalities have increased during the current economic recession because socioeconomically disadvantaged people tend to be most affected by the crisis [19]. To add new evidence to this field, the objective of this study was to analyse trends in socioeconomic inequalities in suicide mortality before (2001–2004 and 2005–2008) and during (2009–2012) the economic recession in two geographical settings in Spain.

## Methods

### Design and study population

This study analyses inequalities in mortality in 3 different periods of time, based on individual data from the Basque Country and Barcelona city, two settings where the information of the mortality register is linked with the census [20]. The Basque Country is one of the 17 main regions of Spain (called Autonomous Communities) located in the north of the country. At the end of 2001 it had 2,082,587 inhabitants, increasing to 2,179,815 by the end of 2011. The unemployment rate was 11.6% in 2001, 7.3% in 2006 and 14.9% in 2011. Barcelona, the second largest city in Spain is located on the north-eastern coast of the Iberian Peninsula and had a population of 1,505,325 inhabitants in 2001 and 1,615,448 in 2011. The unemployment rate was 10.5% in the last trimester of 2001, 5.5% in 2007 and 18.8% in 2012.

We considered the at-risk population to be all residents older than 25 years from 2001 to 2012, and analysed suicide deaths whose underlying cause-of-death included ICD-10 codes X60 to X84 (intentional self-harm). We choose 25 years as a cut-off point because university studies are finished by this age. For both Barcelona and the Basque Country, these deaths were confirmed from forensic sources.

For Barcelona, we included suicide deaths in the mortality register that occurred among residents between 2001 and 2012, and for whom complete educational level information was available (872 men and 424 women in total). The educational level of the deceased was obtained through record linkage between the mortality register and the municipal census of Barcelona. Due to a lack of linkage, we excluded 91 suicide deaths for men (9.4%) and 34 for women (7.4%). Information on the at-risk population was obtained from the local census, and included age, sex and the educational level.

For the Basque Country, we used a population-based census-linked longitudinal mortality study. Individual census records from the 2001 and 2006 population censuses were linked with the mortality registry. Individuals from the 2001 census were followed up from November 2001 (date of the 2001 Census) to October 2006, and those from the 2006 census were followed up from November 2006 to December 2012. Deaths were weighted by the inverse of the proportion of linked cases. The exact number of person-years of follow-up was calculated by subtracting the date of the corresponding census from the date of death (for deceased persons) or from the end date of the follow-up. We included 1225 and 449 suicide deaths among men and women, respectively. The percentages of male and female suicide deaths for whom no information on educational level was available due to a lack of linkage were 9.5% and 5.6% for the 2001–2006 period, and 5.6% and 3.7% for the 2006–2011 period, respectively.

### Variables and indicators analysed

The variables studied were age, sex, education (highest level of education completed) and year of death. Age of death was categorised in three age groups (25–44, 45–64, and ≥65 years). Educational level was categorised in five groups: a) no formal education or pre-primary education (0–4 years of education), b) primary education (5–6 years of education), c) lower secondary education (7–9 years of education), d) upper secondary education (10–14 years of education), and e) university education (15 years or more of education). Year of death was grouped into three time periods, two before the onset of the economic recession (2001–2004 and 2005–2008), and one after the onset of the recession (2009–2012). Although the economic recession actually started during the 4th term of 2008, unemployment levels did not start to rise noticeably until 2009.

### Data analysis

We conducted separate analyses for each geographical setting (Barcelona and Basque Country), for men and women, for the three age groups (25–44, 46–64, and ≥65) as well as for an “all ages” group, and for the three time periods (pre-crisis 2001–2004, pre-crisis 2005–2008 and crisis 2009–2012). We calculated the crude death rates and 95% confidence intervals (95% CI) for each case.

We fit Poisson regression models to obtain the relative index of inequality (RII) of mortality by educational level and the corresponding 95% CI values. The dependent variable was the logarithm of the mortality rate, and the independent variable was educational level as a quantitative variable (with five values between 0 and 1, based on the midpoint of the educational level group’s position in the cumulative educational level distribution of the population). When we analysed “all ages” we also included age-group as independent variable. The RII value can therefore be interpreted as the ratio of mortality rates between the two extremes of the educational spectrum (taking into account all educational levels). Moreover, we adjusted models with the 3 periods together where the independent variables were age group, period, educational level and the interaction between period and educational level in order to know if RII changed over time.

We also estimated absolute differences of mortality between the two extremes of the educational spectrum calculating the Slope Index of Inequality (SII) using a novel approach [21]. First, we obtained different SII scores for each age group using an additive Poisson model, and then calculated the weighted sum of these individual SII scores. The weights applied corresponded to the relative sizes of the age groups in the reference population (in our case either Barcelona or the Basque Country). Where necessary, we corrected for over dispersion in the Poisson models.

## Results

Table 1 shows a descriptive analysis of the total population studied and the number of suicide deaths by sex, age, educational level and setting. In general, the educational level obtained tended to increase overtime, with younger individuals obtaining higher educational levels. Women generally obtained a lower educational level than men, except in the youngest age group.

**Table 1:** Population at risk (Population) and deaths due to suicide (n) by educational level, age group, and sex in the 3 periods. Barcelona and the Basque Country, 2001–2012

			Men						Women					
			Period						Period					
			2001–2004		2005–2008		2009–2012		2001–2004		2005–2008		2009–2012	
Region	Age groups (years)	Educational level	n	Population	n	Population	n	Population	n	Population	n	Population	n	Population
Barcelona	25–44	No education	0	16,761	2	17,597	2	26,534	0	11,484	0	10,662	0	19,976
		Primary education	35	150,244	25	192,988	11	185,215	10	106,469	4	130,452	3	122,275
		Lower secondary	25	219,452	28	218,517	33	213,998	13	194,839	9	182,571	7	153,825
		Upper secondary	39	369,435	24	377,862	28	327,705	20	354,562	19	367,351	19	311,509
		University	10	218,546	19	260,716	18	317,370	9	268,665	4	329,838	13	382,749
	45–64	No education	9	57,484	11	36,027	3	21,435	2	91,244	1	59,465	4	34,873
		Primary education	28	192,740	27	176,852	14	151,645	19	254,107	13	229,281	7	189,267
		Lower secondary	21	130,320	16	143,711	22	154,838	8	174,111	17	191,873	7	199,462
		Upper secondary	15	160,229	30	192,132	29	222,332	9	132,516	9	167,633	18	206,657
		University	18	166,491	22	191,055	23	213,501	8	145,295	13	189,969	13	234,976
	> = 65	No education	34	131,681	27	114,168	23	94,889	23	297,891	15	259,664	7	220,117
		Primary education	44	177,119	38	174,433	32	174,385	17	317,641	22	316,998	20	318,087
		Lower secondary	6	63,523	9	68,540	8	78,062	5	88,296	8	100,550	6	119,014
		Upper secondary	13	71,307	10	76,047	11	84,296	4	63,535	6	71,955	2	83,919
		University	7	71,353	13	78,998	10	95,682	4	49,253	2	56,347	4	71,579
Basque country	25–44	No education	0	6186	0	9166	0	9703	0	5714	1	7046	0	6809
		Primary education	59	309,353	58	354,386	45	299,382	18	267,649	21	273,226	9	207,686
		Lower secondary	25	132,936	18	195,483	23	222,834	3	120,703	2	158,175	5	166,574
		Upper secondary	34	306,533	59	404,437	33	390,974	17	244,539	16	328,524	15	322,248
		University	23	336,946	28	421,956	19	396,831	10	423,977	20	563,919	12	550,963
	45–64	No education	4	27,507	2	25,822	4	18,812	1	37,750	1	35,219	0	23,942
		Primary education	65	401,591	58	487,946	69	457,379	20	515,018	22	614,179	19	543,895
		Lower secondary	13	125,197	22	184,504	23	193,273	11	125,214	11	197,202	14	214,907
		Upper secondary	13	140,419	19	225,183	25	282,539	4	76,290	8	141,946	9	199,518
		University	11	138,346	17	209,706	21	247,102	1	98,588	8	180,154	9	253,002
	> = 65	No education	29	67,467	30	76,330	18	67,008	8	125,060	7	143,903	7	128,391
		Primary education	82	335,552	118	427,315	113	442,520	39	507,242	43	662,113	34	699,452
		Lower secondary	4	27,978	6	50,453	11	74,158	4	28,286	1	55,199	7	83,700
		Upper secondary	5	30,479	4	49,970	9	67,847	0	17,724	0	29,989	4	42,453
		University	2	41,151	7	61,052	1	76,793	1	25,258	2	37,990	2	49,239

**Fig. 1:**
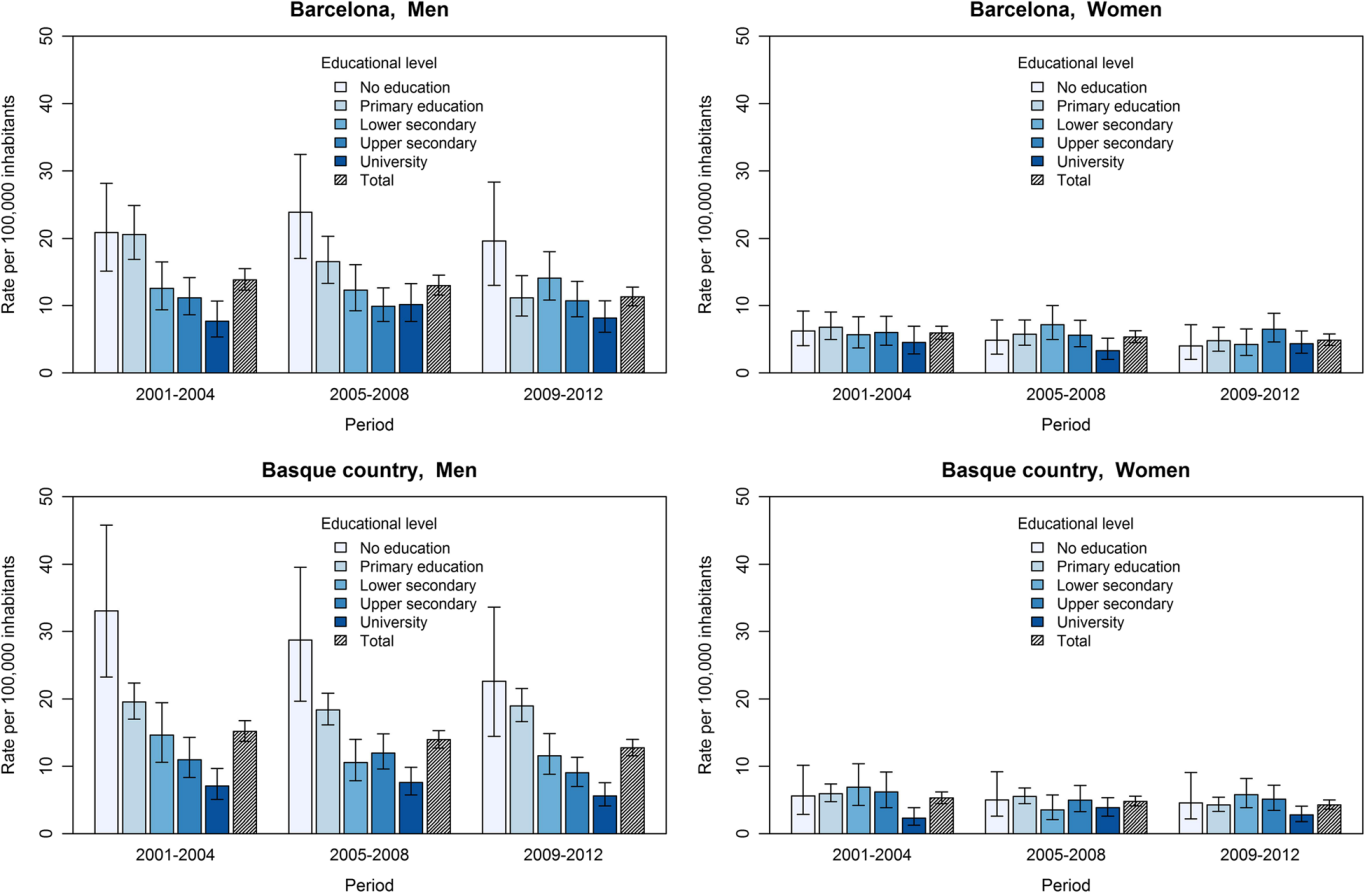
Suicide mortality rates by educational level in Barcelona and the Basque Country for men and women, 2001–2012

Figure 1 shows total suicide death rates by educational level for men and women in Barcelona and the Basque Country. Total suicide mortality rates were higher among men than among women, and showed a more stable pattern. For men, we observe an inverse relationship with educational level in all three time periods. Among women, the rates were lower and did not show such a clear relationship; they showed a similar pattern during all three time periods.

Table 2 shows the relative (RII) and absolute (SII) differences in inequalities between educational levels during the three time periods, for men and women, and for the two geographical settings. For men, there was a clear association between suicide mortality and educational level in all groups. In the Basque Country, most of the RII scores were > 2 for the three time periods and all age groups, and educational level inequalities remained stable. In Barcelona, RII scores were slightly lower, falling to approximately 2 for all three time periods. The SII scores tended to decrease in Barcelona during the time periods studied, but show a U-shaped pattern for the Basque Country. Among men younger than 65 years of age in the Basque Country, RII scores diminished during the second time period, but increased again in the third. For example, the RII score of the 25–44 years age group was 4.04 in 2001–2004, 2.36 in 2004–2008, and 3.96 in 2009–2012; we observed a similar pattern in the 45–64 age group.

**Table 2:** Association of educational level with suicide mortality: Relative Index of Inequality (RII) and Slope Index of Inequality (SII) by age group and sex in the 3 periods. Barcelona and the Basque Country, 2001–2012

			2001–2004				2005–2008				2009–2012			
Region	Sex	Age groups (years)	RII	95% CI	SII	95% CI	RII	95% CI	SII	95%CI	RII	95% CI	SII	95% CI
Barcelona	Men	25–44	4.54	(2.24–9.18)	16.93	(9.36–24.49)	2.98	(1.44–6.16)	8.84	(2.21–15.46)	1.99	(0.72–5.52)	6.33	(−1.74–14.40)
		45–64	1.88	(0.88–3.99)	8.73	(−0.76–18.23)	1.62	(0.72–3.67)	5.58	(−5.17–16.33)	1.01	(0.47–2.17)	0.12	(−9.31–9.56)
		> = 65	2.56	(1.02–6.46)	27.00	(7.27–46.73)	1.72	(0.83–3.56)	12.93	(−0.21–26.07)	2.58	(1.16–5.70)	13.86	(2.54–25.19)
		Total	2.98	(1.82–4.87)	17.46	(11.22–23.70)	2.00	(1.28–3.13)	8.87	(3.40–14.33)	1.67	(1.03–2.71)	5.42	(0.29–10.56)
	Women	25–44	2.76	(1.03–7.42)	6.17	(0.81–11.53)	3.02	(0.68–13.44)	5.37	(0.10–10.64)	1.06	(0.35–3.21)	0.63	(−4.03–5.29)
		45–64	0.71	(0.25–2.06)	−2.59	(−8.84–3.65)	0.77	(0.22–2.69)	−2.10	(−10.29–6.09)	0.66	(0.18–2.37)	−3.95	(−9.98–2.08)
		> = 65	1.23	(0.37–4.11)	1.34	(−6.59–9.27)	0.73	(0.27–1.98)	−1.77	(−8.03–4.48)	0.71	(0.17–2.86)	−3.38	(−10.51–3.76)
		Total	1.24	(0.62–2.48)	2.50	(−1.91–6.90)	1.05	(0.49–2.26)	1.03	(−3.00–5.07)	0.77	(0.35–1.72)	−1.26	(−5.06–2.54)
Basque Country	Men	25–44	4.04	(2.02–8.08)	17.12	(9.24–25.00)	2.36	(1.30–4.31)	10.30	(3.94–16.66)	3.96	(2.03–7.70)	11.95	(6.45–17.44)
		45–64	2.66	(1.23–5.74)	11.62	(2.84–20.40)	1.72	(0.86–3.41)	4.89	(−1.99–11.78)	2.58	(1.12–5.93)	10.44	(0.36–20.53)
		> = 65	4.33	(2.03–9.26)	30.27	(11.65–48.89)	3.62	(1.88–6.97)	35.28	(21.08–49.47)	3.34	(1.66–6.74)	34.44	(23.09–45.79)
		Total	3.96	(2.49–6.28)	19.49	(13.17–25.81)	2.57	(1.74–3.81)	14.44	(9.53–19.36)	3.68	(2.44–5.54)	17.70	(13.49–21.90)
	Women	25–44	3.03	(1.01–9.04)	5.94	(1.03–10.86)	2.43	(0.90–6.58)	3.88	(−0.70–8.46)	2.03	(0.66–6.22)	3.01	(−0.81–6.83)
		45–64	0.58	(0.12–2.81)	−3.71	(−10.04–2.62)	0.50	(0.16–1.58)	−3.38	(−9.66–2.90)	0.57	(0.20–1.63)	−2.76	(−6.93–1.41)
		> = 65	0.85	(0.25–2.83)	−0.18	(−8.34–7.98)	1.42	(0.44–4.59)	1.54	(−3.19–6.28)	0.59	(0.16–2.18)	−2.07	(−9.23–5.09)
		Total	1.48	(0.62–3.53)	2.73	(−1.46–6.91)	1.49	(0.74–3.01)	1.16	(−2.14–4.46)	0.83	(0.37–1.85)	−0.13	(−3.52–3.26)

95% CI: 95% Confidence Intervals

RII value can be interpreted as the ratio of mortality rates between the two extremes of the educational spectrum (taking into account all educational levels). SII is the absolute differences of mortality between the two extremes of the educational spectrum (taking into account all educational levels)

For women, there was an association between educational level and suicide mortality during the first time period in the 25–44 years age group, but not in the other age groups or time periods; this pattern was observed in both geographical settings. The SII scores tended to decrease, and in the Basque Country even reversed.

Models including the interaction between period and educational level always showed that the interaction was not statistically significant (RII did not change over time).

## Discussion and conclusions

This study shows a stable trend in suicide mortality in two Spanish settings, but inequalities according to educational level mainly affected men. Educational level inequalities were stable over the three time periods in both settings (i.e. no increase during the economic recession). These results do not support our hypothesis.

We did not observe an increase in suicide mortality rates among men or women after the economic recession; these findings are consistent with those of other Spanish studies [15, 22, 23]. However, Saurina et al. did find an increase in suicide mortality in a study of Catalan municipalities with more than 10,000 inhabitants, albeit only among women of working age [18]. Moreover, other studies analysing slightly different time periods have also described an increase in suicide mortality [16, 17, 24]. Previous studies did find differences in suicide rates between Spanish provinces, with Barcelona showing low rates [25]. Note that our study is based on two Spanish settings, Barcelona city and the Basque Country, where the economic recession has not had such a drastic effect as in other Autonomous Communities.

Our results, with higher rates of suicide mortality among men, are consistent with those of other studies [5, 6, 26] showing a gender difference in suicide mortality. Moreover, suicide mortality rates increased when educational level decreased, mainly among men. There are several explanations for these gender and socioecionomic inequalities based on an “ecological system” for suicide among men, and mainly of those disadvantaged, including societal influences (e.g. changes in types of employments, unemployment which has increased mainly among men during the economic recession), aspects related to not meeting the standard masculinity (the way men are brought up to behave and the roles, attributes and behaviours that society expects of them) as for example losing the role of breadwinner in the family or the demands of a society with greater equality, as well as individual factors [27].

Relative educational level inequalities in suicide mortality increased in the recession period only among men younger than 65 years of age in the Basque Country. Therefore, this group of men of the Basque Country was the only one that followed our hypothesis, showing how inequalities in suicide mortality increased among men of working age, due to the increase in suicide mortality among those with less educational level. These results could be explained by the increase of unemployment which could affect their role as family breadwinners. But the results found are not completely consistent with the intense economic recession that has affected Spain in recent years. This economic recession has mainly affected the most socioeconomically deprived population [19], and previous research in Spain shows how poor mental health has worsened since its onset. This decline in mental health was mainly observed among men of working age [28] and among population groups who were more affected by the crisis [29], such as those at risk of home eviction [30, 31]. This decline in mental health among less privileged groups in Spain is not reflected in suicide mortality in Barcelona and only partially in the Basque country, probably due to the different impact of the economic crisis in these 2 settings, which has been less important compared to other Spanish regions. Further studies to monitor inequalities in suicide mortality should be continued into the near future.

In other contexts, the available evidence on suicide mortality rates is more disparate. For instance, Cope et al. found that suicide mortality rates in England and Wales were considerably higher among individuals living in more deprived areas. However, there was no evidence to suggest that rates in these areas during the 2008 economic recession increased more markedly than rates in more affluent areas [9]. Additionally, Valkonen et al. described how socioeconomic inequalities in suicide mortality worsened after the economic crisis of the 1990s, with rates and inequalities also being higher among men [32]. Studies in Japan [33, 34] and South Korea [35] have shown that, following the 2008 economic downturn, suicide mortality in men increased more markedly among those who held managerial and professional jobs posts.

Ours is one of the first studies to analyse inequalities in suicide mortality during the current economic recession in Spain. To our advantage, we had access to registries of suicide mortality using forensic information that allowed us to more accurately diagnose the causes of death in both of the geographical settings we studied [36]. However, the low number of suicide deaths in some educational levels and age groups affected the precision of the estimates. As such, using RII and SII scores allowed us to study all educational levels simultaneously. Another limitation of this study is the percentage of deaths excluded due to missing information on educational level, although we do not have any evidence of significant differences in missingness between educational levels, so suicide mortality inequalities should not be affected. It is worth mentioning that we were only able to analyse one indicator of socioeconomic position (educational level), and were not able to obtain data on social class, occupation or income. Educational level has the advantage that it does not change over time, although precisely for this reason it does not capture changes in socioeconomic position [37]. Finally, we have to acknowledge that we included deaths in the “X” series and not “Y” series (uncertain suicides). However, as the code of the causes of death was filled using the forensic information, Y codes are not used in these settings.

This study highlights a stable pattern of socioeconomic inequalities in suicide mortality among men before and during the economic recession in two distinct geographical settings of Spain. However, it is necessary to further monitor this mortality in the future because the economic recession could have greater long-term effects.
