# Correction to: Socioeconomic inequalities in suicide mortality before and after the economic recession in Spain

**DOI:** 10.1186/s12889-019-6687-3

**Published:** 2019-04-01

**Authors:** Carme Borrell, Marc Marí-Dell’Olmo, Mercè Gotsens, Montse Calvo, Maica Rodríguez-Sanz, Xavier Bartoll, Santiago Esnaola

**Affiliations:** 1 Agència de Salut Pública de Barcelona, Plaça Lesseps 1, 08023 Barcelona, Spain; 2 CIBER Epidemiología y Salud Pública (CIBERESP), Madrid, Spain; 3 Institut d’Investigació Biomèdica (IIB Sant Pau), Barcelona, Spain; 4 Universitat Pompeu Fabra, Barcelona, Spain; 5 Department of Health of the Basque Country, Vitoria-Gasteiz, Spain

**Correction to: BMC Public Health**

**https://doi.org/10.1186/s12889-017-4777-7**

It has been highlighted that the original article [1] contained an error in the Funding section. This Correction article shows the incorrect and correct Funding section.

Incorrect:

This article has been partially supported by the project Efectos de la crisis en la salud de la población y sus determinantes en España (PI13/00897), funded by the Instituto de Salud Carlos III.

Correct:

This article has been partially funded by the project Efectos de la crisis en la salud de la población y sus determinantes en España (PI13/00897), funded by the Instituto de Salud Carlos III (co-funded by European Regional Development Fund).
